# A Circular Approach for the Valorization of Tomato By-Product in Biodegradable Injected Materials for Horticulture Sector

**DOI:** 10.3390/polym15040820

**Published:** 2023-02-07

**Authors:** Alain Bourmaud, Kolja Konschak, Coralie Buffet, Méline Calatraba, Anton Loïc Rudolph, Antoine Kervoëlen, Basile Gautherot, Estelle Bonnin, Johnny Beaugrand

**Affiliations:** 1 Université de Bretagne Sud, IRDL UMR CNRS 6027, 56100 Lorient, France; 2 INRAE, UR BIA Biopolymères Interactions Assemblages, Rue de la Géraudière, 44316 Nantes, France; 3 CAVI, Compagnie des Agrafes à Vigne, 3, Rue du Vieux Moulin, 10110 Buxières sur Arce, France

**Keywords:** biopolymer, tomato fiber, morphological analysis, mechanical properties, SEM

## Abstract

This study focuses on the use of tomato (*Solanum lycopersicum* L.) by-product biomass from industrial plants as reinforcement for designing a range of new degradable and biobased thermoplastic materials. As a novel technique, this fully circular approach enables a promising up-cycling of tomato wastes. After an in-depth morphological study of the degree of reinforcement through SEM and dynamic analysis, mechanical characterization was carried out. Our mechanical results demonstrate that this circular approach is of interest for composite applications. Despite their moderate aspect ratio values (between 1.5 and 2), the tomato by-product-reinforced materials can mechanically compete with existing formulations; PBS-Tomato fiber, for example, exhibits mechanical performance very close to that of PP-flax, especially regarding strength (+11%) and elongation at break (+6%). According to the matrix and particle morphology, a large range of products—biobased and/or degradable, depending on the targeted application—can be designed from tomato cultivation by-products.

## 1. Introduction

Due to their good mechanical properties and relatively low weight, plant fiber composites are being used in an ever-increasing number of industrial applications [1]. To develop local, sustainable, and economically viable value chains, the use of agricultural by-products or waste can be of great interest if volumes, reproducibility, and suitable mechanical characteristics can be achieved. In this context, the potential of by-products from flax [2] or other food plants such as rice, wheat, or sunflower have already been successively investigated [3]. Compared to raw polymer materials, the use of plant fibers in composite reinforcement induces a linear increase in the Young’s modulus according to the fiber volume fraction. The tensile strength equally increases significantly, up to a maximal value generally around 30–35%-vol, depending on the quality of the blend and particle morphology [1]. Moreover, the use of plant fibers can be of interest regarding the end-of-life of products, enabling—if degradable polymers are used—the design of fully biodegradable composite materials, and with planned lifetimes [4]. The integration of plant fibers in composite materials can modify the degradation mechanisms of polymers, with plant fiber degradation generally being faster than biodegradable polymer degradation alone as the integration of plant fibers induces the co-existence of two interfacial regions at the fiber–matrix and fiber–fiber scale [5]. It has been reported that these interfacial regions are privileged areas for water and microorganism diffusion inside materials [6,7].

The focus of the present study is the tomato biomass produced by greenhouses, and the fibrous particles thereof, that is potentially usable at the end of cultivation for the reinforcement of composite materials. With more than 16.5 million tons of tomatoes harvested in Europe across an area of 233,000 ha in 2020, the tomato is the nightshade crop with the second largest production in Europe after the potato [8]. While there are approaches for the alternative use of overproduced tomatoes for fresh sale or visually unsatisfactory tomatoes, there are no proposals for the use of the remaining plant by-products, i.e., stems and leaves [8]. The plastic accessories used for greenhouse tomato cultivation amount to approximatively between 500,000 and 700,000 parts per hectare. The vast majority are produced with petro-sourced and non-degradable polymers such as polyolefin polymers. Thus, they impede the composting of the by-products and cause processing costs to be assumed by market gardeners. Due to the high number of accessories, their recycling is utopian. Therefore, the development of more environmentally friendly products is a crucial aspect to address for the future of the chain value.

In this study, the research work deals with the scientific design of new thermoplastic formulations that integrate tomato by-product biomass. In addition to the use of biomass as reinforcement, the choice of polymer matrix also plays a significant role in the sustainability of a fiber composite. The industry standard for the use of natural fibers constitutes petrochemical matrices such as poly-(propylene) (PP). Many studies are dedicated to the development of PP-based plant fiber composites, and their mechanical and recycling potential has been widely demonstrated [9,10,11]. In order to move away from fossil-fuel-based raw materials and reduce their environmental impact, various bio-based polymers have already been researched as matrixes. Of particular interest are the plastics poly-(lactic acid) (PLA), poly-(hydroxyalkanoate) (PHA), and poly-(butylene-succinate) (PBS) [4], which have already proven their potential as plant fiber composite matrixes. For example, Seggiani et al. [12] and Fracz et al. [13] studied the reinforcement of different PHAs with posidonia and wood fibers and highlighted the mechanical potential of the materials developed. PBS has also been widely used with a large variety of plant reinforcements such as flax [14,15,16] or hemp [17]. PLA has been associated with plant fibers for a long time, especially due to its high interfacial compatibility with cellulosic products [4] and excellent mechanical performance [18,19,20]. These polymers have the property of being industrially biodegradable or compostable [4,21]. It has been demonstrated that these polymers show different degradation modes according to the environmental conditions. For example, PLA is generally very sensitive to temperature due to its rubbery state below the transition temperature [22], which induces a rapid decrease in its molecular weight, yet it has a long degradation time. PLA needs at least 150 days to fragment in the soil at a temperature between 15 °C and 25 °C. PHA, in the same conditions, loses 80% of its weight after 50 days, and PBS degrades very slowly in a soil compost at 30 °C, losing only 13.5% of its weight after 80 days [23,24].

This research is dedicated to the development of new, fully degradable composite materials reinforced with tomato plant biomass by-products. After an SEM and morphological study of the fibrous particles of tomato biomass, a set of PBS-reinforced compounds was produced with different particle sizes; mechanical properties will be addressed and compared to a range of polymers and flax composites.

## 2. Materials and Methods

### 2.1. Materials

The plant fibers used in this study were obtained from tomato biomass cultivated in a greenhouse in France in 2019. After harvest, they were dried for 24 h at 60 °C, crushed, and sieved. Accordingly, tomato fibers of sizes <50 μm (T50), 50–100 μm (T100), 100–250 μm (T250), 250–500 μm (T500), and <1000 μm (T1000) were obtained. The flax fibers (*Linum usitatissimum* L., variety Alizé) used for the mechanical comparison between composites were harvested in Normandy in 2018, dew-retted, scutched, hackled, and then carefully cut to a length of 1 mm (Depestele Company, Bourguebus, FR). PP (Total, PPC 10642), PBS (PBI 003, Natureplast, Ifs, France), PHA (PHI 002, Natureplast), and PLA (3001D, Natureworks, Minnetonka, MN, USA) polymers were used. Their melt flow indices (MFI) are 22, 20, 15–30, and 22 g/10 min (2.16 kg, 190 °C), respectively. A compatibilizer (PP-g-MA, OREVAC CA100, Arkema, Colombes, France, MFI 44) was used (3%-wt) with the PP matrix to obtain a modified matrix (MAPP).

### 2.2. Morphological Analysis of Tomato Particles

The morphology of the tomato particles was investigated using a dynamic image analyzer (QICPIC, SympaTec GmbH, Clausthal-Zellerfeld, Germany). According to the expected particle sizes of <50–1000 μm, the M5 lens with a detection range of a minimum of 1.8 μm to a maximum of 3755 μm was used. Approximately 150 mg of fibers was weighed from each sample and placed in suspension with 20 mL of ethanol and 20 mL of distilled and filtered water. To prevent agglomeration of the particles and to allow for the accurate detection of the separate particles, the suspension was stirred with an ultrasonic device. A further 960 mL of distilled and filtered water was then added, and the suspension was kept in motion by means of a magnetic stirring rod. A pump was used to move the suspension through the QICPIC system, wherein it was analyzed and finally pumped back into the measuring beaker in a closed circuit. Depending on the sample, between 56,000 and 3.4 million particles were analyzed. The PAQXOS software (Version 4.2, SympaTec GmbH, Clausthal-Zellerfeld, Germany) was used for evaluation. Due to the non-elongated particle shape, we decided to use the Feret diameter parameters for estimating L, D, and L/D. Feret maximal and minimal diameters were calculated for each particle after consideration of all possible orientations (0° to 180°). Therefore, the maximum and minimum can be significantly larger and smaller than the diameter of the equivalent circle, respectively. Finally, the ratio of L/D was determined for each tomato fraction.

For SEM analysis, tomato particles were taken from each sample and initially sputtered with a thin layer of gold (Edwards Sputter Coater, Atlas Copco Group, Stockholm Sweden). They were then viewed under a scanning electron microscope (Jeol JSM-IT500HR, JEOL Ltd., Akishima, Japan). Images were taken at magnifications of 25×, 100×, and 500× for each sample.

### 2.3. Thermal Analysis of Tomato Particles

Thermo-Gravimetric analysis (TGA) was performed using a TGA2050 (TA Instruments—Waters SAS, Guyancourt, France). Around 2 mg to 5 mg of each sample was placed in a platinum crucible and heated at 10 °C/min from ambient temperature to 700 °C under nitrogen atmosphere. The weight loss was recorded during the heating process and plotted as a function of temperature.

### 2.4. Biochemical Analysis of Tomato Particles

The monosaccharide content of each batch was determined using wet chemical analysis. Approximately 1 g of raw tomato samples was cryoground (SPEX 6700 freezer mill); then, approximately 5 mg of each sample was hydrolyzed first in 12 M H_2_SO_4_ (Sigma Aldrich, Saint-Quentin Fallavier, France) for 30 min at 25 °C in a heating block, and then the molarity was diluted to 1.0 M H_2_SO_4_ in presence of inositol as internal standard and subsequently heated for 2 h at 100 °C. Liquid–gas chromatography (Perkin Elmer, Clarus 580, Shelton, CT, USA) was performed at 205 °C with H_2_ as carrier gas in order to analyze the alditol acetate derivatives of the individual neutral monosaccharides (arabinose, rhamnose, glucose, xylose, galactose, and mannose) [25]. The automated detection of m-hydroxybiphenyl was applied to determine the galacturonic acid (GalA) and glucuronic acid (GlcA) concentrations, which were combined to form uronic acid (UA). Each monosaccharide content was summed to obtain the total monosaccharide content, given as the percentage in weight of dry matter mass. Measures were performed in triplicates and calibration was performed with three known concentrations of standard monosaccharide solutions. The lignin content was quantified from the same cryoground particles by colorimetrical analysis following the acetyl bromide method [26] on approx. 20 mg per essay. The chemicals were laboratory-grade from Sigma Aldrich and the analyses were performed in at least three independent assays, with lignin expressed as the weight % of the dry matter mass.

### 2.5. Composite Materials Processing and Mechanical Testing

Before processing, all materials were dried in an oven under vacuum at 60 °C for at least 12 h. A co-rotating twin screw extruder (FSCM 21–40, TSA, Cernobbio, Italy) with a screw diameter of 20 mm and an L/D ratio of 40 was used for extrusion. The temperature varied depending on the polymer used (PBS: 140 °C, PHA: 160 °C, PLA: 170 °C, and PP and MAPP: 180 °C). The rotational speed was 300 rpm. First, PBS was combined with all tomato biomass fractions (T50, T100, T250, T500, and T1000) with a fiber weight fraction of 30%. Then, a comparison of matrixes (PBS, PHA, PLA, and PP-g-MA) was performed using the T1000 fraction (30%-wt). The material obtained was then granulated (T5, Meccanoplastica, Castiglione Olona, Italy) and dried again at 60 °C for at least 12 h before injection molding of ISO 527 dog bone specimens (HM 80/120 E, Battenfeld Kunststoffmaschinen,, Kottingbrunn, Austria).

Finally, the injected specimens were mechanically characterized in a tensile test according to ISO 527-2 with an MTS Synergie 1000RT (MTS System Corporation, Eden Prairie, MN, USA) machine in a controlled environment (23 °C and 48% RH). The test specimens were previously equilibrated for at least 12 h within the controlled environment. The speed of the tensile test was set to 1 mm/min. The force applied to the test specimen was measured with a 10 kN sensor, and the deformation of the test specimen during the tensile test was measured with an extensometer (MTS System Corporation, Eden Prairie, MN, USA) attached to the specimen (initial length 25 mm). 

## 3. Results

### 3.1. Investigation of Tomato Particle Morphology, Thermal Behavior, and Biochemical Composition

**Figure 1:**
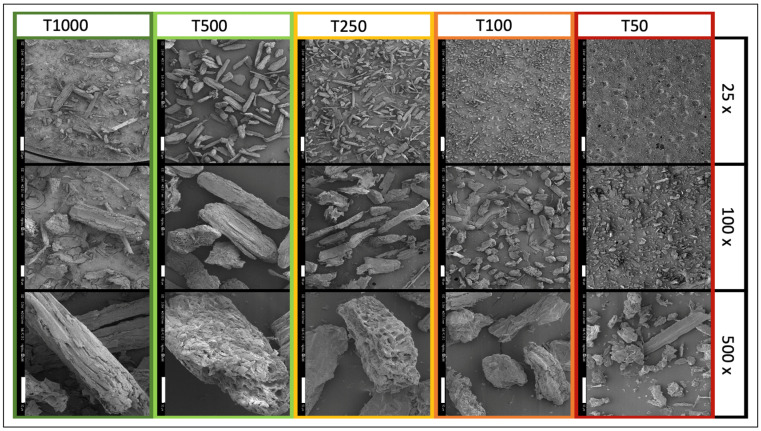
SEM observations of tomato biomass particles at different magnifications. For 25×, 100×, and 500× magnification, the white scale bar corresponds to 500, 100, and 50 mm, respectively.

**Figure 2:**
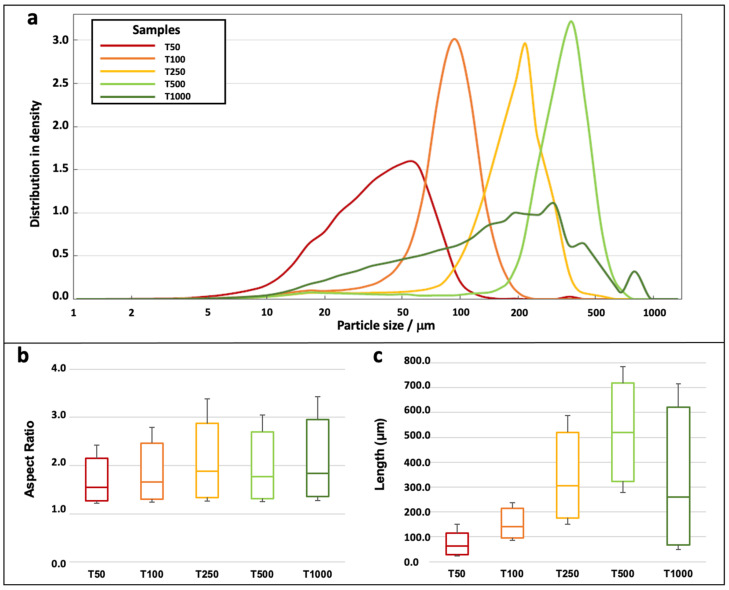
Morphological analysis of tomato biomass particles with particle size distribution (**a**), L/D aspect ratio (**b**), and length (**c**) values for T50, T100, T250, T500, and T1000 fractions.

Figure 1 and Figure 2 show SEM observations at different scales and a morphological analysis of the five tomato biomass sizes, respectively. 

As expected, the SEM images show a strong difference in size between the samples. The particles of the T50 sample, which is very clearly visible under 100× magnification, are clearly smaller than the particles of the following samples. It is also noticeable that the homogeneity of the particle sizes is lowest in the T1000 sample; a mix of large particles with a low aspect ratio that are thin and elongated, and small, dust-like particles can be seen. 

In addition, the difference in the length/diameter aspect ratios becomes clear and was confirmed by morphological analysis (Figure 2b), with a progressive increase in L/D corresponding to the size of the particles; one can notice that the T1000 sample covers the widest range of aspect ratios. The relative volumetric distribution of the particle sizes is shown in Figure 2a. Apart from the T1000 sample, which can be considered a mix of the other batches with the possible presence of leaf or pedunculi, which have a different grinding behavior, the samples show a uniform bell-shaped progression of probabilities, indicating a normal distribution of the samples. The T1000 sample has several local minima and maxima and spans a much wider range of particle sizes than the rest of the samples. The average fiber lengths (Figure 2b) are 83 μm, 166 μm, 314 μm, 507 μm, and 250 μm for the T50, T100, T250, T500, and T1000 batches; they are in line with the targeted values. Thus, these different fractions cover a large range of morphologies, which are different in terms of homogeneity and aspect ratio; the impact of these parameters on the composites’ performance will be assessed in the next section.

**Figure 3:**
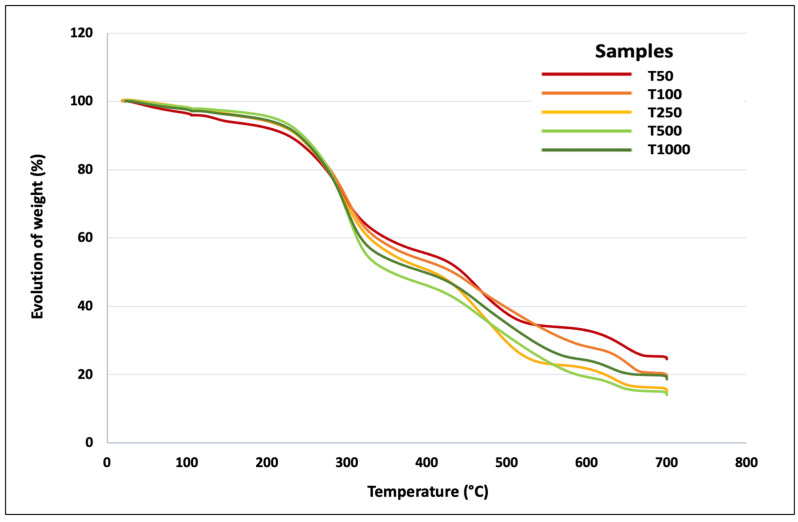
Thermal behavior of the different fractions of tomato particles.

The thermal properties of the different tomato particles were investigated in order to anticipate their behavior during a process cycle, which can induce high temperatures when a thermoplastic matrix is used. Table 1 summarizes the main thermal properties of the five fractions of tomato particles and Figure 3 shows the corresponding weight–temperature curves.

The thermal characteristics of the different fractions of tomato particles do not show major variations. Regarding water loss, it is between 2% and 3% for all fractions; only the T50 fraction shows a higher level of water loss, namely, 4.1%. This can be explained by a higher specific surface of the particles and by an easier access to the porous structure of the tomato particles. The temperatures of the three degradation peaks, generally attributed to non-cellulosic polymers, cellulose, and lignins [27], are very close regardless of the fraction considered. The main difference between the samples is the ash content, which decreases progressively for batches T50 to T500. The value attributed to lot T1000 is intermediate, which is logical since this lot is an assembly of the other fractions. This high ash content for the finest batches demonstrates the significant presence of mineral residues, probably soil, after the various sieving phases. This point is a potential scientific and technological bottleneck for future compounding operations and should be considered in future pilot-scale tests.

The chemical composition of the tomato plant by-product was also investigated. The obtained sugar and lignin concentrations are shown in Table 2. 

The lignin content in all tomato samples is between 11.9 ± 0.1% and 14.0 ± 0.6%. The lignin fraction increases progressively with sample size, reaching a maximum for the T500 batch; this observation is consistent with the thermal behavior of the polymers, which shows a progressive shift of peak 3, attributed to lignin degradation, towards higher temperatures. This lignin fraction is important compared to bast fibers (generally between 1 and 3% for flax fibers) and relatively low if wood fibers are considered (around 30%) [28]. It is important to keep in mind that the fractions analyzed are a mix of the different tissues of the plant; among them, conduction cells are probably more lignin-reinforced due to their involvement in sap conduction. A significant lignin concentration, such as this one, is generally interesting in terms of milling efficiency [29]. The literature reports concentrations between 5 and 14% for lignin according to the lot materials [30], whereas another study on post-harvest tomato plant stems [31] found less lignin content, at around 8%. In this study, we have obtained results in the same range, as the samples have about 14% lignin content for the less-refined particle sizes, which corresponds to the raw material. 

Regarding the samples’ monosaccharidic composition, one can notice significant differences between T50, T100, and T250 on the one hand and between T500 and T1000 on the other hand. For these two sets of samples, the glucose and xylose content are significantly higher and statistically different for the T500 and T1000 samples. This indicates that segregation into cellular types occurs when the sieving fineness increases. One can see a clear cutoff between T250 and T500 regarding the xylose content (from 3.3% to 6.18%, respectively) and the glucose content (from 13.4% to 20.6%), indicating that milling refines the cellular or histological type recovered from the tomato plants. The glucose content is a good indicator of cellulose content and is also generally well correlated with the mechanical performance of cell walls; these results give a first indication about the mechanical potential of the different fractions. The T500 and T100 samples appear to be the most promising batches.

If we refer to the recent literature data regarding tomato plant by-product polysaccharides, interestingly, the cellulose content reported is about 30 to 40% [30,31]. In this study, our raw material (T1000) is 20% *w*/*w*, which is slightly less. In contrast, the hemicellulose content reported is about 4 to 11% [30,31], whereas we obtained about 18% (sum of the non-glycosidic monosaccharides), which is slightly more. 

We hypothesize that the method of quantification could be partially responsible for this difference, since, in this study, a direct quantification of the cellulose monomer was performed, whereas the other study used an indirect method based on sequential extraction and gravimetric measurements such as Van Soest. Overall, the comparison of our results with earlier investigations suggests that the variation induced by varietal or agronomic practices do not drastically modify the biochemical composition of tomato plant by-products. 

### 3.2. Mechanical Performance of PBS–Tomato Injected Compounds

**Figure 4:**
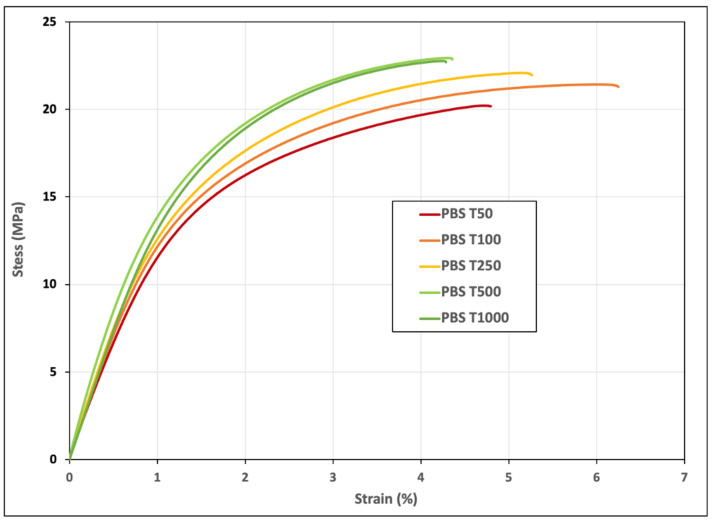
Stress–Strain behavior of PBS–tomato biomass composite materials.

**Figure 5:**
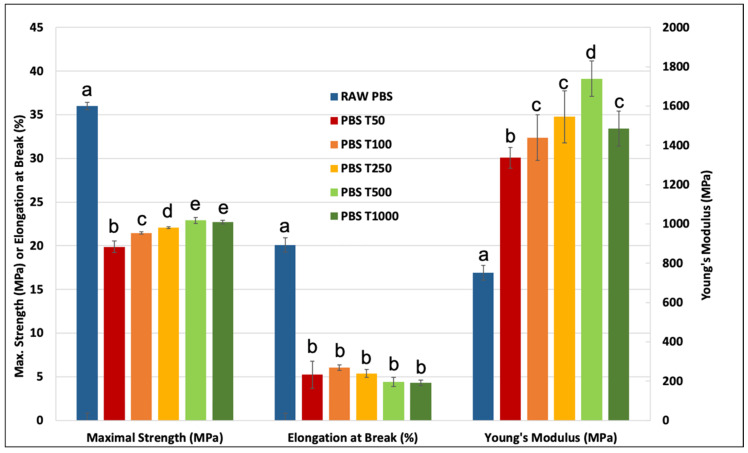
Tensile mechanical properties of PBS–tomato biomass composite materials. Letters indicate the statistical differences between the mechanical values. When letters are different, the property is significantly different.

The mechanical behavior and properties of the different PBS–tomato particle composite materials are shown in Figure 4 and Figure 5, respectively. For both tensile strength and Young’s modulus, a progressive increasing trend can be seen with a greater fiber length up to T500. The composite with T1000 as a reinforcement shows a significant decrease in Young’s modulus but a stability of strength at break.

The Young’s modulus of composite materials is generally impacted by both the length and the aspect ratio of fiber reinforcement [32]. The observed decrease in the T1000 sample can be linked to the previous morphological observations and especially to the wide length distribution of the sample. Despite this decrease, one can notice that all the modulus values are significantly higher than the raw matrix stiffness, with an improvement reaching +131% and +103% for the T1000 and T500 reinforced compounds, respectively. This demonstrates the potential of the reinforcement of tomato particles in terms of stiffness and also the good compatibility of PBS with plant cell walls, which are generally considered hydrophilic, as already addressed in a previous work [16]. As expected, the elongation at break decreases with the increase in the mean fiber length (Figure 5). An exception in this regard is the T50 sample with the shortest fibers, though a relatively large standard deviation can be seen in this case. Moreover, no statistical difference can be demonstrated for the strain-at-break values between the batches. Nevertheless, the strain values are high compared to typical biobased composite materials [16].

The maximal strength is penalized by the heterogeneity and possibly poor intrinsic cohesion of the plant reinforcements, which induces early composite breakage. However, the tensile stress increases progressively from batches T50 to T500 with statistically different values, which confirms the potential, from a mechanical point of view, of using relatively large fibers.

**Figure 6:**
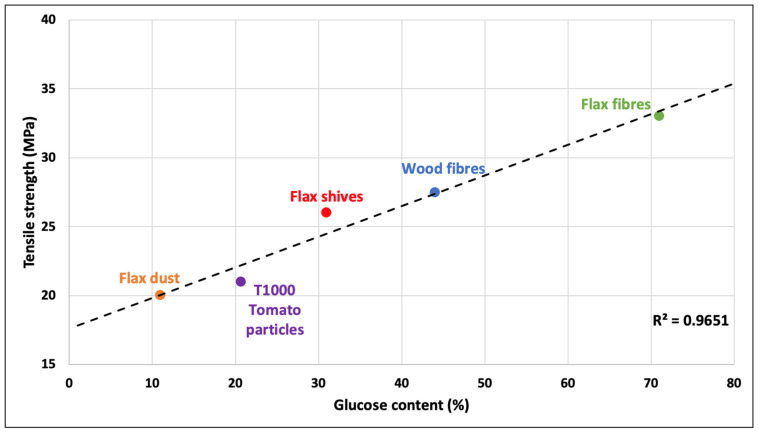
Correlation between the tensile strength and the glucose content of the reinforcement. All the blends have been produced with the same polymer (PP-g-MA) and same fiber weight fraction (30%) [2].

Nevertheless, the best strength values are obtained for the two samples with the highest cellulose content. This demonstrates the impact of the cellulose content on the mechanical performance of plant fiber composites, as evidenced in Figure 6, designed with a set of literature data and the results from this work (with PP-g-MA and T1000 particles).

Indeed, of all the constituents of plant walls, cellulose has the best mechanical properties [33]. Flax fibers, which contain the highest fraction of cellulose (around 70%), enable the acquirement of composite materials with high tensile strength. These composites are stronger than the pure matrix, reaching a strength of 18.7 GPa for PP (in this work) [28]. This is also the case for wood fibers and flax shives [28]; these reinforcing particles both originate from the xylem of plants and are thus significantly less rich in cellulose but much more rich in lignin. Despite this intermediate cellulose content, they contribute significantly to the reinforcement of composites, with strength values that are still higher than those of the polymer matrix, and these reinforcements can be used to configure materials with average properties yet advantageous production costs. In terms of behavior and biochemical composition, tomato particles are similar to flax dust, which was studied in a previous work [2]. In this case, it is more of a filler than a reinforcement; nevertheless, these biomass resources can be used to develop materials that do not require excellent mechanical properties but can be produced at very low cost. For example, they can be used as a substitute for chalk. With the same PP matrix, this latter material exhibits equivalent tensile strength [14].

### 3.3. Comparison with a Range of Polymer Matrixes

**Figure 7:**
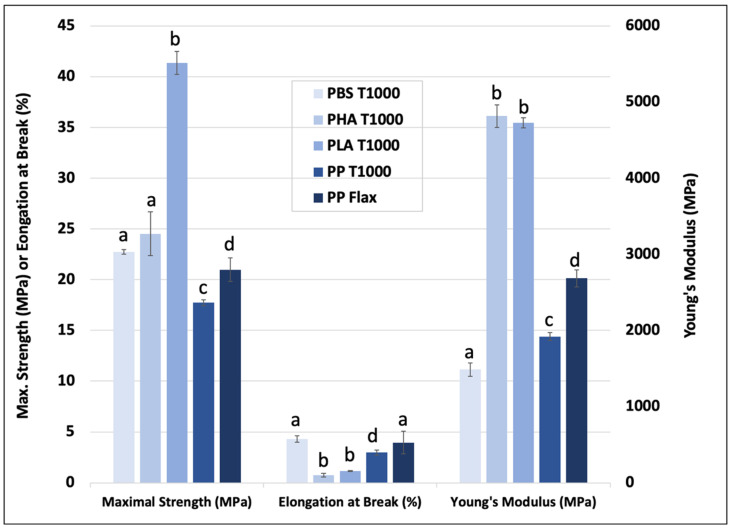
Comparative tensile mechanical properties of PBS–tomato biomass particle composite materials. Letters indicate the statistical differences between the mechanical values. When letters are different, the property is significantly different.

Figure 7 presents, for the same tomato fiber weight fractions (30%), a comparison between PBS-T1000 and the PLA-, PP-, and PHA-T1000 compounds. A PP–Flax fiber compound is added as reference [14]. The T1000 fraction was selected for its industrial interest, representing a good compromise between mechanical performance (Figure 3), economical, and environmental cost. In terms of stress at break, all the formulations reinforced with tomato particles are competitive with the PP–Flax used in different industrial sectors, especially the automotive industry. 

The results are more contrasted in terms of Young’s modulus because, in this case, the morphology of the particles and their mechanical performance are particularly impactful, and the rigidity of the matrix also plays a major role. 

**Figure 8:**
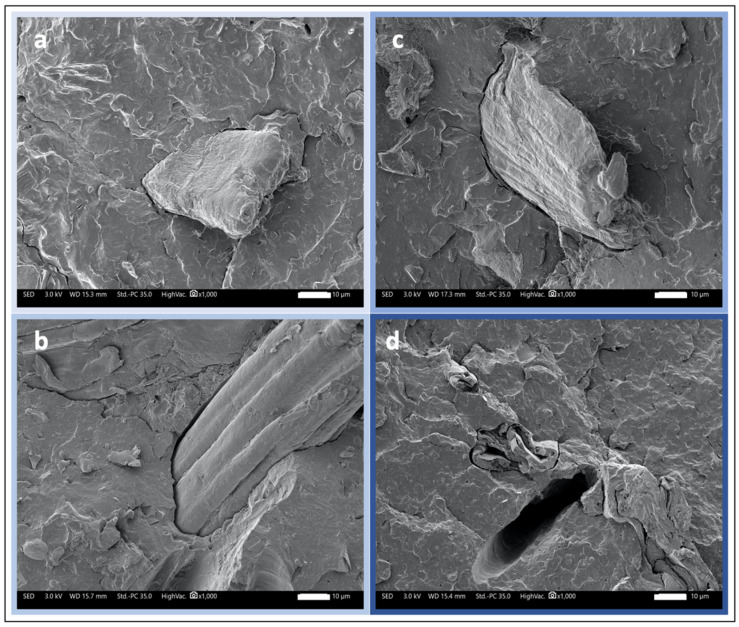
SEM images of fiber-matrix interface for PBS (**a**), PHA (**b**), PLA (**c**), and PP (**d**) tomato reinforced composite materials.

The images shown in Figure 8 provide a good impression of the quality of the interfaces for each of the four polymers used with tomato fibers. The PBS matrix seems to show the best interfaces with a very reduced gap between the matrix and the fiber. Then, the quality of the bonding between PLA and PHA on the one hand and the tomato fiber on the other hand seems to be roughly equivalent, also showing good interface quality. Finally, the situation is different with respect to PP. Specifically, matrix loosening, such as that shown in the figure, is observed; this demonstrates the weak bond between the flax fibers and the PP matrix.

These results can be compared with the literature values obtained from composites made by injection from similar matrices and different plant reinforcements (Table 3). For example, Seggiani et al. [12] and Fracz et al. [13] studied the reinforcement of different PHAs with posidonia and wood fibers with similar weight ratios to what we used; the properties obtained were between 2.3 GPa (Posidonia) and 6.1 GPa (wood) for the tensile modulus and between 21.0 MPa and 30.7 MPa for strength. Our PHA-T1000 values are intermediate, showing the suitability of tomato fibers in terms of competing with some medium-quality bio-masses. Regarding the PBS matrix, the literature values [14] obtained with flax fibers are higher than what we were able to measure with tomato particles (about 2.8 GPa for the modulus of the flax PBS and 35 MPa for its stress). This confirms the results of the measurements of the aspect ratio and cellulose content in the tomato particles. The reinforcement potential of these particles is lower than that of fibers with much better mechanical and morphological performance. As far as PLA is concerned, there is a great deal of data in the literature with similar weight ratios to the one we used here. For example, the mechanical properties obtained with PLA–flax [16] or PLA–wood [20] are superior to ours, with stress values of around 55 MPa compared to the 41.4 MPa that we were able to obtain. The tensile moduli are also higher, with tensile moduli of 7.4 and 6.2 GPa for PLA–flax and PLA–wood, respectively.

However, these new materials offer a range of performance in terms of stiffness, which is generally inversely correlated with the elongation-at-break values. This allows mixes to be configured to suit the desired applications and functions.

## 4. Conclusions

Thanks to an original circular approach to transforming the plant biomass by-product into the injected composite, our study demonstrated the possibility of using tomato industry by-products to design new high-performing and degradable eco-composite materials. 

The main scientific conclusions of the study are as follows:

- The different batches of tomato particles that we were able to study show low aspect ratios, generally around two, but a good dispersion of their morphology.
- A small variation in the chemical composition of the batches of particles could be demonstrated, which shows a selectivity of the tissues to grinding; moreover, the relative lignin content increases progressively with the particle size, which translates into a slight delay in thermal degradation.
- Composite materials made from a PBS matrix and crushed tomato particles have quite acceptable mechanical properties, which increase steadily with particle size, reaching their maximum for batches with an average length of 500 µm.
- The cellulose content of the tomato particles provides an intermediate mechanical strength for the composites considered. These data confirm an existing and already demonstrated correlation between the cellulose content and the maximum stress of injected polypropylene matrix composites.
- Finally, by combining these tomato particles with a range of biodegradable matrices, we were able to demonstrate that they provide intermediate-level material properties; in most cases, the tomato particles act as a reinforcement and not just a filler.

According to the morphology of the reinforcements and that of the polymer matrix, it is feasible to design a large range of injected materials with good stiffness properties and photosynthetic carbon storage potential and that are competitive with flax-reinforced references, especially in terms of strain at break and stiffness. The use of the PBS matrix offered a choice of end-of-life characteristics, i.e., recycling or degradation, and, mechanically speaking, appears to be a good compromise when softness is targeted. It appears that the composite materials developed here can mechanically compete with low-value plant fiber reinforcements or chalk. In order to fully validate these formulations, in the near future, it will be necessary to continue this work by studying the biodegradation of the materials developed.

## Figures and Tables

**Table 1:** Thermal properties of the tomato plant particle samples.

	T50	T100	T250	T500	T1000
Water content (%)	4.1	2.5	2.6	2.1	2.6
Peak 1 (°C)	205	205	204	205	205
Peak 2 (°C)	405	405	405	405	405
Peak 3 (°C)	607	608	606	610	610
Ash (% dry matter)	24.9	19.8	15.5	14.5	19.2

**Table 2:** Biochemical composition of the tomato plant particle samples. All lignin and monosaccharide concentrations are expressed in percentage of dry matter.

	T50	T100	T250	T500	T1000
Lignin (%)	11.9 ± 0.1 ^b^	13.8 ± 0.3 ^a^	12.6 ± 0.2 ^a^	14.0 ± 0.6 ^a^	13.4 ± 0.4 ^a^
Arabinose (%)	1.20 ± 0.10 ^a^	1.35 ± 0.06 ^a^	1.20 ± 0.12 ^b^	1.13 ± 0.11 ^a^	1.50 ± 0.13 ^a^
Rhamnose (%)	0.51 ± 0.06 ^a^	0.78 ± 0.10 ^b^	0.52 ± 0.05 ^a^	0.54 ± 0.15 ^a^	0.64 ± 0.05 ^a^
Galactose (%)	1.66 ± 0.50 ^a^	1.83 ± 0.38 ^a^	1.63 ± 0.15 ^a^	1.54 ± 0.08 ^a^	1.52 ± 0.02 ^a^
Glucose (%)	11.5 ± 0.66 ^b^	13.3 ± 0.27 ^b^	13.4 ± 1.35 ^b^	20.1 ± 5.01 ^a^	20.6 ± 0.65 ^a^
Xylose (%)	2.49 ± 0.35 ^b^	3.16 ± 0.04 ^b^	3.30 ± 0.56 ^b^	6.18 ± 1.96 ^a^	5.99 ± 0.24 ^a^
Mannose (%)	1.03 ± 0.23 ^a^	1.14 ± 0.19 ^a^	1.20 ± 0.19 ^a^	0.96 ± 0.08 ^b^	1.60 ± 0.21 ^a^
Uronic Acid (%)	6.80 ± 0.22 ^b^	7.77 ± 0.48 ^a^	7.69 ± 0.13 ^a^	8.11 ± 0.50 ^c^	7.91 ± 0.26 ^a^

Values sharing the same letter within a line are not statistically different at the 0.05 level of confidence.

**Table 3:** Comparison of the mechanical properties of plant fiber composites of this study with literature values. We selected materials processed by compounding and injection molding and having the same fiber weight fraction (30%).

	Young’s Modulus (GPa)	Maximal Strenght (MPa)	Elongation at Break (%)	Reference
PBS T1000	1.49 ± 0.09	22.7 ± 0.2	4.3 ± 0.3	This study
PBS flax fibers	2.81 ± 0.11	34.7 ± 1.2	2.3 ± 0.2	[14]
PBS chalk	0.95 ± 0.06	25.8 ± 0.9	3.7 ± 0.5	[14]
PHA T1000	4.82 ± 0.15	24.5 ± 2.1	0.8 ± 0.2	This study
PHA posidonia fibers	2.32 ± 0.15	21.0 ± 2.7	2.4 ± 0.5	[12]
PHA wood fibers	6.11 ± 0.36	30.7 ± 0.8	1.1 ± 0.1	[13]
PLA T1000	4.72 ± 0.07	41.4 ± 1.1	1.2 ± 0.4	This study
PLA flax fibers	7.44 ± 0.02	55.4 ± 1.2	1.4 ± 0.1	[16]
PLA wood fibers	6.23 ± 0.05	54.3 ± 1.1	8.7 ± 0.4	[20]

## Data Availability

Data supporting the findings of this study are available on simple request from the corresponding author.
